# Mapping movement, mood, motivation and mentation in the subthalamic nucleus

**DOI:** 10.1098/rsos.171177

**Published:** 2018-07-18

**Authors:** Amritha Gourisankar, Sarah A. Eisenstein, Nicholas T. Trapp, Jonathan M. Koller, Meghan C. Campbell, Mwiza Ushe, Joel S. Perlmutter, Tamara Hershey, Kevin J. Black

**Affiliations:** 1 Department of Biomedical Engineering, Washington University in St. Louis, St. Louis, MO 63130, USA; 2 Department of Psychiatry, Washington University School of Medicine, St. Louis, MO 63110, USA; 3 Department of Radiology, Washington University School of Medicine, St. Louis, MO 63110, USA; 4 Department of Neurology, Washington University School of Medicine, St. Louis, MO 63110, USA; 5 Department of Neuroscience, Washington University School of Medicine, St. Louis, MO 63110, USA; 6 Department of Occupational Therapy, Washington University School of Medicine, St. Louis, MO 63110, USA; 7 Department of Physical Therapy, Washington University School of Medicine, St. Louis, MO 63110, USA

**Keywords:** subthalamic nucleus, deep brain stimulation, Parkinson's disease, emotions, inhibition (psychology), short-term memory

## Abstract

The anatomical connections of the subthalamic nucleus (STN) have driven hypotheses about its functional anatomy, including the hypothesis that the precise anatomical location of STN deep brain stimulation (DBS) contributes to the variability of motor and non-motor responses across patients with Parkinson's disease (PD). We previously tested the hypothesis using a three-dimensional (3D) statistical method to interpret the acute effects of unilateral DBS at each patient's clinically optimized DBS settings and active contact. Here, we report a similar analysis from a new study in which DBS parameters were standardized and DBS locations were chosen blind to clinical response. In 74 individuals with PD and STN DBS, STN contacts were selected near the dorsal and ventral borders of the STN contralateral to the more affected side of the body. Participants were tested off PD medications in each of three unilateral DBS conditions (ventral STN DBS, dorsal STN DBS and DBS off) for acute effects on mood, apathy, working memory, response inhibition and motor function. Voltage, frequency and pulse width were standardized, and participants and raters were blind to condition. In a categorical analysis, both dorsal and ventral STN DBS improved mean motor function without affecting cognitive measures. Ventral STN DBS induced greater improvement in rigidity and anxiety than dorsal STN DBS. In the 3D analysis, contact location was significant for body hypokinesia, rigidity and resting tremor, with the greatest improvement occurring with DBS in dorsal STN and zona incerta. The 3D results provide new, direct functional evidence for the anatomically derived model of STN, in which motor function is best represented in dorsal STN. However, our data suggest that functional segregation between motor and non-motor areas of the STN is limited, because locations that induced improvements in motor function and mood overlapped substantially.

## 1. Introduction

Parkinson's disease (PD) is the second most common neurodegenerative disease [1]. PD varies in its presentation; symptoms may include disturbed sleep, depressive symptoms, apathy and cognitive complications in addition to classic motor features such as bradykinesia, rigidity and tremor [2]. Deep brain stimulation (DBS) of the subthalamic nucleus (STN DBS) can improve many of the motor symptoms [3], but changes in mood, motivation and cognition also occur and may be either beneficial or detrimental to the patient [4]. In fact, clinical results vary substantially among patients. Some evidence suggests that the location of stimulation within or around the STN may contribute to the motor, mood and cognitive effects of STN DBS, given its relatively segregated anatomical connections to motor, somatosensory and limbic neural circuits [5]. However, the methods used to test this hypothesis in the past have had limitations, including not examining the entire relevant volume of the brain [6–8], not determining the statistical significance of relationships between behaviour and DBS site [9–12], or not correcting for type 1 errors due to the multiple comparisons inherent in three-dimensional (3D) statistical maps with many data points (i.e. voxels) [13]. Some studies examined the effects of DBS on neuronal response with reference to the volume of tissue predicted to be activated based on electrical field models [14]. We combined the anatomical location of the stimulated electrode with clinical data to produce statistical images that demonstrate DBS locations associated with improvement and worsening of each measured symptom, and determined overall statistical significance from these images using a permutation approach [15]. This method avoids the issues noted above, and identifies whether location relates to clinical response in a statistically rigorous manner controlled for multiple comparisons.

Using this method, we previously examined the acute effects of unilateral STN DBS in PD, using each person's clinically optimized stimulation parameters and electrode contacts. Mood, cognition and motor function were assessed with DBS OFF and ON at least 8 h after the most recent dose of PD-related medication. The 3D analyses suggested that location of stimulation was significantly associated with mood, cognition and some motor outcomes [15]. Most motor measures improved with DBS everywhere in the STN, while a few motor, cognitive and mood measures differed depending on the location of stimulation. A limitation of that study was that stimulation parameters (e.g. voltage) differed across individuals, which could differentially impact behaviour. The stimulation parameters used and the contact chosen were determined through the clinical programming process, so the results could not distinguish whether all participants would have had similar motor benefit with DBS anywhere in the STN, or whether the ideal DBS location simply varied by participant. Therefore, in this new study, all participants with PD had separate, blinded, unilateral stimulation conditions at both dorsal *and* ventral STN locations chosen by brain imaging blind to clinical results. All stimulation parameters were maintained across condition and participant. We hypothesized that our findings would be qualitatively similar to those in our previous report, but that effects might be more striking due to the consistent stimulation parameters and the more uniform approach to selecting DBS locations in both dorsal and ventral STN.

## 2. Material and methods

### 2.1. Participants

Seventy-four patients with PD were recruited through the Movement Disorders Center at Washington University St. Louis School of Medicine (WUSM), St. Louis, MO, USA. Inclusion criteria included bilateral STN DBS therapy for clinically definite PD, as previously defined [16] based on established criteria [17,18]. Patients waited at least 3 months after DBS implantation to participate in the study. Exclusion criteria included neurological conditions such as history of stroke; history of serious head injury (any neurological sequelae, open skull fracture or hospitalization); history of definite encephalitis or oculogyric crises; drug-induced parkinsonism; sustained remission from PD; strictly unilateral features after 3 years; supranuclear gaze palsy; cerebellar signs (ataxia of gait or limbs, central nystagmus, scanning dysarthria or truncal ataxia); early severe autonomic involvement; early severe dementia (within the first year of onset) with disturbances of memory, language and praxis; extensor plantar reflex; Mini Mental State Examination score less than 24 [16]; any defect on brain imaging (such as infarcts, brain tumour, hydrocephalus or congenital defects like lissencephaly but not cavum septum pellucidum); or MPTP(1-methyl-4-phenyl-1,2,3,6-tetrahydropyridine) exposure, for which patients were screened prior to DBS surgery. The demographics of the participants of the study are shown in table 1.

**Table 1.** Demographics and clinical characteristics of 74 research participants with PD. CD-LD, carbidopa–levodopa; CD-LD ER, carbidopa–levodopa extended release; DA, dopamine; MAO, monoamine oxidase; COMT, catechol-*O*-methyl transferase, UDPRS, Unified Parkinson Disease Rating Scale.

	mean (s.d., range)
age (years)	62 (9.1, 43–80)
education (years)^a^	15.1 (2.7, 10–20)
disease duration (years)	12.4 (5.1, 0.51–26.5)
time since STN DBS surgery (months)	18.2 (16.1, 3–77)

	distribution
sex	50 male, 24 female
ethnic origin^b^	65 white, 4 Native American/Alaskan Native, 1 African American, 1 Asian, 2 unknown/other
more affected side, by UPDRS III subscore	41 right, 33 left
dominant hand^b^	65 right, 7 left, 1 ambidextrous
current PD medication^c^^,^^d^	74 CD-LD, 12 CD-LD ER, 33 DA agonist^e^, 7 MAO inhibitor, 32 COMT inhibitor, 25 benzodiazepines, 40 amantadine, 7 antidepressants^f^, 21 other drugs

^a^Four participants missing data.

^b^One participant missing data.

^c^Prior to abstinence on the day of study.

^d^Participant may appear in more than one medication category.

^e^No participant was taking extended release formulations of DA agonists.

^f^Amitriptyline, bupropion, duloxetine, nortriptyline, trazodone.

### 2.2. Subthalamic nucleus deep brain stimulation electrode contact selection

The side of the brain contralateral to the more affected side of the body was stimulated. The more affected side of the body was defined by the side of the body that had higher Unified Parkinson Disease Rating Scale (UPDRS) scores in the off-medication, off-stimulation state [6]. The DBS electrode contacts for each individual were placed in atlas space using a validated method [19,20] to identify the contact locations with respect to the STN. Dorsal and ventral STN DBS contacts were chosen for each participant based on the examination of their position in atlas space. Specifically, a contact within 2 mm of the ventral STN border was chosen as the ventral contact, and a contact within 2 mm of the dorsal STN border was chosen as the dorsal contact, ideally with one unused contact in between [6].

### 2.3. Stimulation protocol

Participants stopped PD medications at midnight before the morning of the study. The UPDRS ratings and mood and cognitive tasks were completed during separate dorsal, ventral and OFF STN DBS sessions over the course of one day. The order of the dorsal, ventral and OFF sessions was randomized and blinded to the participants and raters. The voltage, frequency and pulse width were 2.5 V, 185 Hz and 60 µs, respectively, for most participants. However, 14 participants experienced side effects from 2.5 V and so the voltage was reduced to 1.6–2.3 V.

### 2.4. Measurements

Motor symptoms were rated with the UPDRS, part III-motor, administered by a trained clinician blind to stimulation condition. UPDRS subscale scores for bradykinesia (sum of scores from finger taps, hand movements, rapid movement of hands and leg agility), rigidity, tremor at rest and total were summed contralateral to the stimulated side of the brain. The UPDRS ‘body bradykinesia and hypokinesia' item score was considered separately (hereinafter ‘body').

Cognition was evaluated via the spatial delayed response (SDR) and the Go/No-Go (GNG) tasks. The SDR task assesses short-term and working memory for spatial information, and was performed as described previously; the variable of interest was the distance between actual and recalled (after a 15 s delay) cue locations, or error [21,22]. The GNG task assessed the ability to select and inhibit a pre-potent motor response appropriately under conditions of high pre-potent response strength [23], and was performed as described previously [6]. The discriminability index, Pr, was the outcome measure, defined as the proportion of hits minus the proportion of false alarms. Only data from participants who reached a criterion of Pr > 0.5 in the OFF DBS condition were included in the analyses.

Self-rated current affective state was assessed using visual analogue scales (VASs) based on the circumplex model of emotion [24] and transformed to valence and arousal scores, as described previously [15,25]. Separate scores for anxiety and apathy were also measured using a VAS [8]. Higher scores on valence, anxiety and apathy represented, respectively, happier, less anxious and less apathetic states.

### 2.5. Primary statistical analyses

#### 2.5.1. Outliers

In the datasets for all measures in both statistical analyses—univariate and 3D—outliers were defined as data values more than 3 s.d. from the mean. The datasets and statistical outcomes shown are based on the datasets with these outliers removed.

#### 2.5.2. Univariate statistics

Dorsal, ventral and OFF DBS scores for each measure, including total contralateral UPDRS, tremor at rest, rigidity, bradykinesia, SDR error in mm, GNG Pr, valence, arousal, apathy and anxiety, were compared using separate repeated-measures ANOVAs. If the ANOVA *p*-value was statistically significant, dorsal-OFF, ventral-OFF and dorsal–ventral difference scores for the corresponding measures were compared with 0 using one-sample *t*-tests. We repeated the ANOVAs for participants who received 2.5 V STN DBS, excluding participants who received less than 2.5 V STN DBS. The threshold for statistical significance for ANOVAs was *α* = 0.005, reflecting Bonferroni correction for multiple comparisons (0.05/10 comparisons). The threshold for statistical significance for one-sample *t*-tests was *α* = 0.05.

#### 2.5.3. Statistical mapping of deep brain stimulation effects to subthalamic nucleus anatomy

Our mapping method is described in detail in Eisenstein *et al*. [15]. Briefly, four statistical maps were generated for each measure. (i) An *N* image shows the number of stimulated contacts that contributed dorsal or ventral DBS difference scores to each voxel of the map, i.e. within 1.3 mm. Voxels with *N *< 6 were not included in further steps. (ii) A weighted mean image, containing the weighted mean difference scores across participants, with nearer contacts weighted higher. (iii) A *t* image depicting weighted *t* values derived from single-sample *t* tests comparing the mean difference scores (dorsal–OFF or ventral–OFF) at each voxel with zero. (iv) A *p*-image containing *p*-values for the *t* test at each voxel. We repeated the statistical mapping for participants who received 2.5 V STN DBS, excluding participants who received less than 2.5 V STN DBS.

#### 2.5.4. Type 1 error correction for multiple comparisons and sample bias

To test whether the anatomical location of the active DBS contact significantly contributed to clinical effects, we used a permutation test as previously described [15]. Briefly, for each measure, a summary score reflecting the extent and amplitude of significant voxels in the *p*-image was generated, and compared with 1000 summary scores generated similarly but from randomly chosen pairings of the active contact locations and difference scores. We considered a *p*-value ≤ 0.05 (i.e. a summary score that would place it in the top 50 of the 1000 random data permutations) to indicate that DBS location significantly contributed to a measure's difference scores. We repeated type 1 error correction for participants who received 2.5 V STN DBS, excluding participants who received less than 2.5 V STN DBS.

## 3. Results

### 3.1. Distribution of contacts

**Figure 1.**
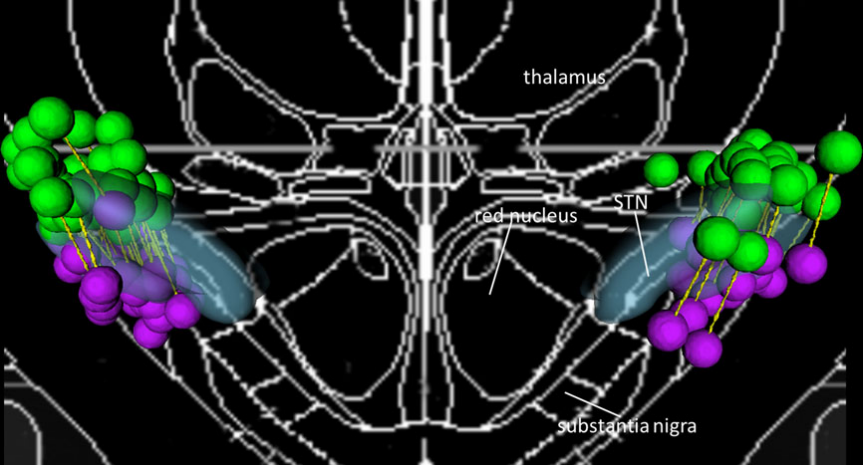
Distribution of contacts included in the analyses shown as green (dorsal) and purple (ventral) spheres, with paired contacts of each participant indicated by yellow connecting rods, and blue transparent regions indicating the subthalamic nucleus (STN).

The stimulated contacts from 74 participants, each with a dorsal STN and ventral STN contact, are shown in figure 1. All contacts were located within 2 mm of the STN border.

### 3.2. Univariate results

The effects of dorsal or ventral STN DBS on mood, cognitive and motor measures (irrespective of 3D active contact location) are described in table 2. Ventral or dorsal DBS significantly improved all UPDRS motor scores, anxiety, valence and apathy. Unilateral STN DBS did not significantly affect the mean scores for the GNG and SDR cognition tests. Dorsal scores differed significantly from ventral scores for anxiety and rigidity, which both improved more with ventral STN DBS than with dorsal STN DBS. Results were similar when participants stimulated at less than 2.5 V were excluded from analyses, except that the univariate effects of dorsal STN DBS did not differ significantly from those of ventral STN DBS on any measure (electronic supplementary material, table 1).

**Table 2.** Outcome measures, by STN DBS conditions and DBS site (dorsal versus ventral STN).

rmANOVA result		mean difference (s.d.)	d.f.	sig. (two-tailed)
	*mood and motivation*^a^			
*F*_2,138_ = 13.7, *p* < 0.001*	anxiety: dorsal versus OFF	6.2 (16.0)	69	*0.002*
	anxiety: ventral versus OFF	9.5 (17.0)	69	*<0.001*
	anxiety: dorsal versus ventral	−3.3 (12.9)	69	*0.04*
*F*_2,138_ = 1.9, *p* = 0.15	arousal: dorsal versus OFF	0 (0.2)		
	arousal: ventral versus OFF	−0.03 (0.2)	N/A	N/A
	arousal: dorsal versus ventral	0.03 (0.1)		
*F*_2,138_ = 11.1, *p* < 0.001*	valence: dorsal versus OFF	0.1 (0.2)	69	*0.001*
	valence: ventral versus OFF	0.2 (0.3)	69	<*0.001*
	valence: dorsal versus ventral	−0.05 (0.2)	69	0.12
*F*_2,138_ = 4.8, *p* =* 0.009*	apathy: dorsal versus OFF	7.1 (20.8)	69	*0.006*
	apathy: ventral versus OFF	6.3 (23.7)	69	*0.03*
	apathy: dorsal versus ventral	0.8 (17.9)	69	0.7
	*cognition*^b^			
*F*_2,132_ = 1.5, *p* = 0.2	GNG: dorsal versus OFF	0.03 (0.2)	N/A	N/A
	GNG: ventral versus OFF	0.01 (0.2)		
	GNG: dorsal versus ventral	0.02 (0.1)		
*F*_2,136_ = 1.4, *p* = 0.2	SDR: dorsal versus OFF	−1.0 (8.5)	N/A	N/A
	SDR: ventral versus OFF	−1.3 (9.3)		
	SDR: dorsal versus ventral	0.33 (9.31)		
	*movement*^c^			
*F*_2,138_ = 30.9, *p* < 0.001*	bradykinesia: dorsal versus OFF	−1.6 (2.1)	69	<*0.001*
	bradykinesia: ventral versus OFF	−1.8 (2.2)	69	<*0.001*
	bradykinesia: dorsal versus ventral	0.2 (1.9)	69	0.8
*F*_2,140_ = 27.1, *p* < 0.001*	body: dorsal versus OFF	−0.5 (0.6)	70	*<0.001*
	body: ventral versus OFF	−0.5 (0.6)	70	*<0.001*
	body: dorsal versus ventral	−0.01 (0.6)	70	0.5
*F*_2,140_ = 38.5, *p* < 0.001*	rigidity: dorsal versus OFF	−0.8 (1.1)	70	*<0.001*
	rigidity: ventral versus OFF	−1.1 (1.2)	70	*<0.001*
	rigidity: dorsal versus ventral	0.3 (0.9)	70	*0.006*
*F*_2,140_ = 30.2, *p* < 0.001*	tremor at rest: dorsal versus OFF	−1.2 (1.6)	70	*<0.001*
	tremor at rest: ventral versus OFF	−1.2 (1.8)	70	*<0.001*
	tremor at rest: dorsal versus ventral	−0.01 (1.1)	70	0.9
*F*_2,140_ = 75.3, *p* < 0.001*	UPDRS: total dorsal versus OFF	−4.2 (3.6)	70	*<0.001*
	UPDRS: total ventral versus OFF	−4.2 (3.5)	70	*<0.001*
	UPDRS: total dorsal versus ventral	0.02 (2.7)	70	1.0

^a^Four VAS participants were statistical outliers and were omitted.

^b^One GNG and 1 SDR participant were outliers and were omitted.

^c^Three UPDRS participants were outliers and were omitted.

*p-value survives multiple comparison correction (Bonferroni, *α* = 0.005). rmANOVA, repeated-measures ANOVA. All subjects with missing/incomplete data in any measure were removed. Numbers in italics are all *p*-values less than 0.05.

### 3.3. Subthalamic nucleus deep brain stimulation effects depend on deep brain stimulation site

**Figure 2.**
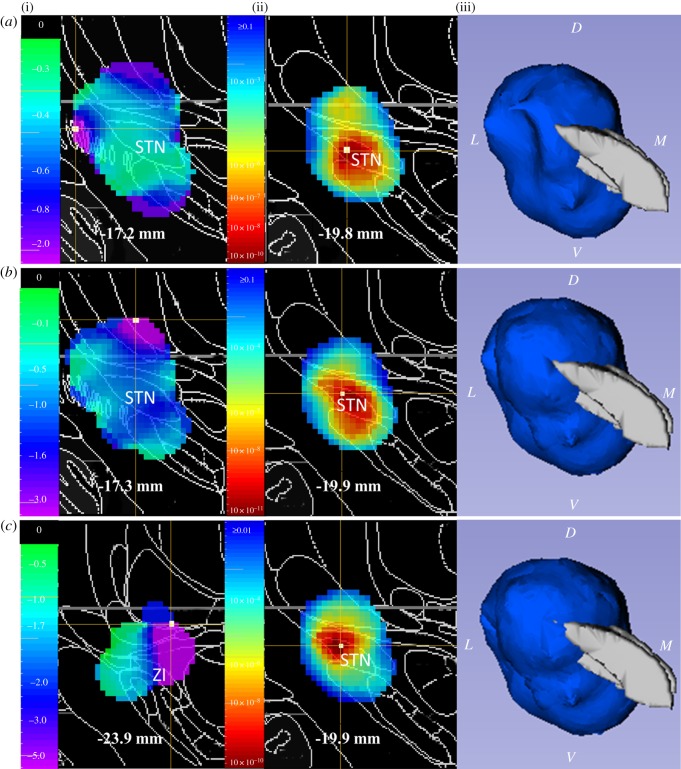
Weighted mean image (i), *p*-image (ii) and 3D *p*-image (iii) for measures with significant effect of contact location in the 3D analyses. (*a*) Body bradykinesia and hypokinesia item. (*b*) Rigidity. (*c*) Tremor at rest. For the weighted mean images, the cooler shades indicate where, on average, the difference scores (ventral-OFF and dorsal-OFF) are more negative (improvement relative to OFF, for motor measures). For the 2D *p*-image, warmer shades indicate more significant *p*-values, while the cooler shades indicate less significant *p*-values. White squares indicate peak coordinates. The 3D image is shown as viewed from anteriorly, and the blue volume indicates values less than 0.05 in the *p*-image. STN, subthalamic nucleus; ZI, zona incerta, D, dorsal, V, ventral, L, lateral, M, medial.

For the analysis based on 3D location of DBS, statistical significance for each measure is shown in table 3. DBS location significantly contributed to the effects of STN DBS on body, rigidity and tremor at rest. Statistical maps for these effects are shown in figure 2. Results were similar after excluding participants who could not tolerate 2.5 V DBS.

**Table 3.** Statistical summary of 3D analyses.^a^

	*p* (permu­tation)	peak weighted mean value	peak weighted mean location (*x*, *y*, *z*)	peak weighted mean location	peak in *p*-image	peak *p*-location (*x*, *y*, *z*)	peak *p*-location
*movement*^b^							
bradykinesia	0.07	−6.5	18, −16.5, −2.5	comb bundle/cp	<0.001	(12.5, −17.5, −3)	dorsal STN
body	0.002	−2.0	(18, −16.5, −2.5)	comb bundle/cp	<0.001	(13, −20, −4)	dorsal STN
rigidity	0.018	−3.0	(12, −16.5, 3.5)	VLPI	<0.001	(12.5, −19.5, −4)	STN
tremor at rest	0.009	−5.0	(8.5, −24, −1.5)	CM	<0.001	(13, −20.5, −3.5)	dorsal STN
UPDRS total	0.07	−10.5	(7, −21, −6)	red nucleus/PBP	<0.001	(12, −20.5, −3.5)	ZI
*mood and motivation*^c^							
anxiety	0.70						
arousal	0.11						
valence	0.28						
apathy	0.61						
*cognition*^d^							
GNG	0.65						
SDR	0.4						

^a^Peak *p* and weighted mean values and locations are only listed for the measures found to be significant in the permutation analysis.

^b^Seventy-one participants contributed to the analyses for the motor measures.

^c^Seventy participants contributed to the analyses for the mood measures.

^d^Sixty-seven and 69 participants contributed to the analyses for the cognitive measures of GNG and SDR, respectively.

STN, subthalamic nucleus; cp, cerebral peduncle; VLPI, ventral lateral posterior thalamic nucleus, internal part; ZI, zona incerta; CM, centromedian thalamic nucleus; PBP, parabrachial pigmented nucleus; GNG, Go/No-Go; SDR, spatial delayed response.

## 4. Discussion

The results support the conclusion that 3D electrode contact location contributes to the motor effects of STN DBS. The peak *p*-values for DBS-induced improvements in motor function were located more dorsally in the STN. This confirms our findings in a different sample, using a different experimental design [15], which showed greater motor improvement in dorsolateral STN, particularly for tremor at rest. Similarly, previous studies also suggested greater improvement in motor function in dorsal STN and the zona incerta (ZI) [10,26,27]. These results fit with anatomical data placing the dorsolateral portion of the STN in a loop connecting primary motor cortex to putamen and motor thalamus, and linking the zona incerta to motor and limbic systems.

In the current study, electrode contact site, as a 3D variable, did not significantly alter the effect of STN DBS on cognitive or mood function in PD. However, ventral STN stimulation improved anxiety more than dorsal STN stimulation in the univariate analysis. A previous study [28] showed increased mood improvement with STN DBS in those with anxiety or mood disorders or higher symptom severity, but psychiatric diagnosis was not assessed in the present study. The non-significant association of contact location and cognitive function is surprising given the present sample size and our previous findings that DBS effects on cognitive measures were location dependent [6,15]. However, there are several differences between the current study and the most comparable previous study [15]. First, the previous study's ON sessions tested participants with their individually optimized DBS settings, including choice of active contact. In other words, in that study the contact selection was not chosen blind to clinical response. Furthermore, because in that study the contacts and settings were optimized clinically, cognitive or affective responses may have contributed to selecting contact or pulse settings that were more likely to improve mood or thinking than the anatomically chosen DBS contacts and standardized pulse settings in the present study.

The strengths of this study include its relatively large sample size, acute stimulation paradigm, assessment blind to the location stimulated and innovative statistical approach. Limitations include the fact that clinical DBS electrode implantation targets the dorsal posterolateral STN, which necessarily limits the number of contacts that fall in the anterior or medial–ventral STN. The limited number of data points in these regions reflects this reality, reducing power in parts of the ventromedial and anterior STN. Second, in some conditions, participants or examiners may have detected when STN was turned on. However, as the focus of the study was the correlation of effect with DBS site, and neither the participants nor the examiners knew the precise locations of the contacts, the study was still blinded for the key variable under investigation, i.e. the location of the active contact. Third, the minimum interval between DBS changes (42 min) was chosen based on previous experience with motor signs, but there may be longer-term effects—on mood and cognitive function in particular—that this investigation may have missed. However, the time limit on the OFF session was also chosen with ethical and practical considerations in mind that preclude extending the time to study more delayed effects. Finally, statistically significant changes in rating scales may not imply syndromal or clinically significant changes. Nevertheless, UPDRS ratings are standard measures of parkinsonian severity, and VASs offer one of the few practicable options for frequent assessment of emotional state. Self-report VAS ratings have correlated well with clinical ratings of depression and anxiety severity [29–32].

Our previous study in a different sample of patients with PD did not support complete functional segregation within the STN of mood, motor and cognitive function [15]. This new sample provides some functional evidence for a dorsal–ventral, motor–non-motor gradient of benefit, in that the 3D analysis found significant location effects for body, rigidity and tremor at rest (table 3), with the evidence for improvement stronger in ZI or dorsolateral STN for these measures (table 3 and figure 2). By contrast, anxiety improved significantly more with ventral than dorsal stimulation (table 2). On the other hand, rigidity also improved more with ventral than dorsal stimulation (table 2); stimulation of either ventral or dorsal STN improved motor function, anxiety, valence and apathy; and cognitive effects were observed with neither stimulation site. Therefore, the direct, functional evidence supports only a mild dorsal–ventral gradient for motor and non-motor effects of STN DBS, rather than a strict dorsal–ventral functional segregation.

## Supplementary Material

Table S1

## Supplementary Material

Table S2

## Supplementary Material

Table S3

## Data accessibility

The data for this study are deposited at Dryad: (http://dx.doi.org/10.5061/dryad.qq934j2) [33].
