# Supplementary material for: Mapping movement, mood, motivation and mentation in the subthalamic nucleus
Source: R Soc Open Sci. 2018 Jul 18;5(7):171177. doi: 10.1098/rsos.171177 (PMC6083651; doi:10.1098/rsos.171177)
Supplement: Table S1 [file rsos171177supp1.docx]

| **Supplementary Table 1.** Outcome measures, by STN DBS conditions and DBS site (dorsal *vs.* ventral STN), excluding participants who received < 2.5V STN DBS. |
| --- |

| **rmANOVA Result** | **Outcome measure** | **Mean difference (S.D.)** | **d.f.** | **Sig. (2-tailed)** |
| --- | --- | --- | --- | --- |
|  | **Mood and motivation** |  |  |  |
| *F_2,114_*=12.9, *p*<**0.001*** | Anxiety: Dorsal vs. OFF  Anxiety: Ventral vs. OFF  Anxiety: Dorsal vs. Ventral | 7.5 (16.1)  9.6 (16.4)  −2.1 (12.5) | 57  57  57 | **0.001**  **<0.001**  0.2 |
|  |  |  |  |  |
| *F_2,114_*=1.9, *p*=0.16 | Arousal: Dorsal vs. OFF  Arousal: Ventral vs. OFF  Arousal: Dorsal vs. Ventral | 0 (0.2)  −0.04 (0.2)  0.03 (0.1) | N/A | N/A |
|  |  |  |  |  |
| *F_2,114_*=9.1, *p*<**0.001*** | Valence: Dorsal vs. OFF  Valence: Ventral vs. OFF  Valence: Dorsal vs. Ventral | 0.1 (0.3)  0.1 (0.3)  −0.03 (0.2) | 57  57  57 | **0.001**  **0.001**  0.33 |
|  |  |  |  |  |
| *F_2,114_*=4.3, *p*=**0.016** | Apathy: Dorsal vs. OFF  Apathy: Ventral vs. OFF  Apathy: Dorsal vs. Ventral | 7.3 (22.0)  6.8 (23.8)  0.6 (17.3) | 57  57  57 | **0.01**  **0.04**  0.8 |
|  |  |  |  |  |
|  | **Cognition** |  |  |  |
| *F_2,106_*=1.1, *p*=0.3 | GNG: Dorsal vs. OFF  GNG: Ventral vs. OFF  GNG: Dorsal vs. Ventral | 0.03 (0.2)  0.02 (0.2)  0.02 (0.2) | N/A | N/A |
|  |  |  |  |  |
| *F_2,112_*=1.4, *p*=0.2 | SDR: Dorsal vs. OFF  SDR: Ventral vs. OFF  SDR: Dorsal vs. Ventral | −1.7 (8.5)  −1.5 (8.3)  -0.16 (8.3) | N/A | N/A |
|  |  |  |  |  |
|  | **Movement** |  |  |  |
| *F_2,112_*=25.1, *p*<**0.001*** | Bradykinesia: Dorsal vs. OFF  Bradykinesia: Ventral vs. OFF  Bradykinesia: Dorsal vs. Ventral | -4.2 (3.8)  -4.2 (3.6)  0 (2.8) | 56  56  56 | **<0.001**  **<0.001**  0.2 |
|  |  |  |  |  |
| *F_2,114_*=21.5, *p*<**0.001*** | Body: Dorsal vs. OFF  Body: Ventral vs. OFF  Body: Dorsal vs. Ventral | −0.5 (0.7)  −0.5 (0.6)  −0.02 (0.6) | 57  57  57 | **<0.001**  **<0.001**  0.8 |
|  |  |  |  |  |
| *F_2,114_*=27.8, *p*<**0.001*** | Rigidity: Dorsal vs. OFF  Rigidity: Ventral vs. OFF  Rigidity: Dorsal vs. Ventral | −0.8 (1.1)  −1.0 (1.2)  0.2 (0.8) | 57  57  57 | **<0.001**  **<0.001**  0.06 |
|  |  |  |  |  |
| *F_2,114_*=28.0, *p*<**0.001*** | Tremor at Rest: Dorsal vs. OFF  Tremor at Rest: Ventral vs. OFF  Tremor at Rest: Dorsal vs. Ventral | −1.3 (1.7)  −1.3 (1.8)  −0.03 (1.1) | 57  57  57 | **<0.001**  **<0.001**  0.8 |
|  |  |  |  |  |
| *F_2,114_*=59.0, *p*<**0.001*** | UPDRS: Total Dorsal vs. OFF  UPDRS: Total Ventral vs. OFF  UPDRS: Total Dorsal vs. Ventral | −4.2 (3.8)  −4.2 (3.6)  0.03 (2.8) | 57  57  57 | **<0.001**  **<0.001**  0.9 |

| *****, *p-*value survives multiple comparison correction (Bonferroni, α = 0.005). rmANOVA = repeated measures ANOVA. All subjects with missing/incomplete data in any measure were removed. |
| --- |
