# Supplementary material for: Mapping movement, mood, motivation and mentation in the subthalamic nucleus
Source: R Soc Open Sci. 2018 Jul 18;5(7):171177. doi: 10.1098/rsos.171177 (PMC6083651; doi:10.1098/rsos.171177)
Supplement: Table S2 [file rsos171177supp2.docx]

| **Supplementary Table 2.** Statistical summary of 3D analyses excluding participants who received < 2.5V STN DBS  ^^^ | | | | | | | |  |
| --- | --- | --- | --- | --- | --- | --- | --- | --- |
|  | *p* (permu­tation) | Peak Weighted Mean Value | Peak Weighted Mean Location  (x, y, z) | Peak Weighted Mean Location | Peak in *p* image | Peak *p* Location  (x, y, z) | Peak *p* Location | |
| **Movement^a^** |  |  |  |  |  |  |  | |
| Bradykinesia | 0.76 |  |  |  |  |  |  | |
| Body | 0.001 | −2.0 | (18, −16.5, −2.5) | comb bundle/‌cp | <0.001 | (12, −17.5, 0) | VLPE | |
| Rigidity | 0.023 | −3.0 | (13, −24, −1.0) | VPM | <0.001 | (13.5, −20, −3) | Dorsal STN/ZI | |
| Tremor at rest | 0.009 | −5.0 | (17, −20, 0) | VPL/eml | <0.001 | (13, −20, −3) | Dorsal STN/ZI | |
| UPDRS total | 0.08 | −10.5 | (7.5, -20.5, -5) | crt | <0.001 | (13, −20.5, −4) | Dorsal STN | |
|  |  |  |  |  |  |  |  | |
| **Mood and motivation^b^** | |  |  |  |  |  |  | |
| Anxiety | 0.77 |  |  |  |  |  |  | |
| Arousal | 0.26 |  |  |  |  |  |  | |
| Valence | 0.46 |  |  |  |  |  |  | |
|  |  |  |  |  |  |  |  | |
| Apathy | 0.67 |  |  |  |  |  |  | |
|  |  |  |  |  |  |  |  | |
| **Cognition^c^** |  |  |  |  |  |  |  | |
| GNG | 0.49 |  |  |  |  |  |  | |
| SDR | 0.49 |  |  |  |  |  |  | |
| ^, Peak *p* and weighted mean values and locations are only listed for the measures found to be significant in the permutation analysis. STN, subthalamic nucleus; cp, cerebral peduncle; VLPE, ventral lateral posterior thalamic nucleus, external part; VPM, ventral posterior medial thalamic nucleus; ZI, zona incerta; VPL, ventral posterior lateral thalamic nucleus; eml, external medullary lamina of thalamus; crt, cerebello-rubro-thalamic fibers; GNG, Go-NoGo; SDR, spatial delayed response. | | | | | | | |  |
