# Supplementary material for: Mapping movement, mood, motivation and mentation in the subthalamic nucleus
Source: R Soc Open Sci. 2018 Jul 18;5(7):171177. doi: 10.1098/rsos.171177 (PMC6083651; doi:10.1098/rsos.171177)
Supplement: Table S3 [file rsos171177supp3.docx]

**Supplementary Table 3: Instructions and anchor points for visual analogue scale ratings.** For each rating, the anchor points are the words included in single quotes.

**Anxiety** (*see Campbell et al 2012 for details on how the ‘anxiety’ score was constructed from these 3 item scores*)

Please rate the way you feel at this moment, from 'very nervous' to 'very calm'.

Please rate the way you feel at this moment, from 'very distressed' to 'very relaxed'.

Please rate the way you feel at this moment, from 'very tense or agitated' to 'very calm or relaxed'.

**Apathy**

Now I will ask about how apathetic or motivated you feel right now. Please rate the way you feel at this moment, from 'very apathetic' to 'very motivated'.

**Valence and Arousal** (*see Limsoontarakul et al 2011 and Eisenstein et al 2014 for details on how ‘valence’ and ‘arousal’ scores were constructed from these 8 item scores*)

Please rate the way you feel at this moment, from 'very sad' to 'very happy'.

Please rate the way you feel at this moment, from 'very grouchy' to 'very cheerful'.

Please rate the way you feel at this moment, from 'very nervous' to 'very calm'.

Please rate the way you feel at this moment, from 'very distressed' to 'very relaxed'.

Please rate the way you feel at this moment, from 'very sluggish' to 'very lively'.

Please rate the way you feel at this moment, from 'very dull' to 'very excited'.

Please rate the way you feel at this moment, from 'very intense' to 'very tranquil'.

Please rate the way you feel at this moment, from 'very aroused' to 'very passive'.

**Supplementary References**

Campbell, M. C., Black, K. J., Weaver, P. M., Lugar, H. M., Videen, T. O., Tabbal, S. D., Karimi, M., Perlmutter, J. S., Hershey, T. (2012). Mood response to deep brain stimulation of the subthalamic nucleus in Parkinson disease. J Neuropsychiatry Clin Neurosci, *24*(1), 28–36. doi:10.1176/appi.neuropsych.11030060

Eisenstein, S. A., Koller, J. M., Black, K. D., Campbell, M. C., Lugar, H. M., Ushe, M., Tabbal, S. D., Karimi, M., Hershey, T., Perlmutter, J. S., Black, K. J. (2014). Functional anatomy of subthalamic nucleus stimulation in Parkinson disease. *Annals of Neurology*, *76*(2), 279–295. doi:10.1002/ana.24204

Limsoontarakul S, Campbell MC, Black KJ, A perfusion MRI study of emotional valence and arousal in Parkinson’s disease. Parkinson’s Dis. 2011;2011:742907. doi: 10.4061/2011/742907
